# Coleções não europeias do antigo Museu de Antropologia da Faculdade de Ciências da Universidade do Porto

**DOI:** 10.1590/S0104-59702025000100044

**Published:** 2025-09-29

**Authors:** Rita Neves Gaspar

**Affiliations:** i Curadora, Museu de História Natural e da Ciência da Universidade do Porto. Porto – Portugal. rgaspar@mhnc.up.pt

**Keywords:** Museu universitário, Coleções não europeias, Biografia dos objetos, Proveniências, University museum, Non-European collections, Biography of objects, Provenance

## Abstract

As coleções do antigo Museu de Antropologia da Faculdade de Ciências, criado em 1912, encontram-se integradas no Museu de História Natural e da Ciência da Universidade do Porto. Com cerca de 125 mil objetos (arqueológicos, etnográficos, de antropologia biológica e numismática), o museu inclui algumas coleções de origem não europeia. Este trabalho pretende identificar as dinâmicas de incorporação das coleções etnográficas não europeias (cerca de 1.100 objetos) ao longo do século XX. Para tal, recorre-se ao levantamento das biografias dos objetos e coleções, identificando proveniências e atores associados, por meio das fontes documentais disponíveis. A identificação dessas dinâmicas de incorporação e documentação dos objetos possibilita vislumbrar como as mudanças de paradigma científico são vertidas para a organização das coleções nas instituições de memória.

## Abstract (en)

The collections of the former Anthropology Museum at the Faculty of Science (established in 1912) are now part of the Natural History and Science Museum at the University of Porto. The museum contains 125,000 objects in archaeology, ethnography, biological anthropology and numismatics, including some collections of non-European origin. This study investigates how the non-European ethnographic collections (around 1,100 objects) were incorporated throughout the twentieth century, examining documentary sources and surveying biographies of the objects and collections to identify provenance and associated actors. Exploring how objects were incorporated and documented allows us to glimpse how changes in the scientific paradigm reflect in the organization of collections in memory institutions.

As coleções não europeias têm hoje um papel central nos museus europeus. A necessidade de contextualizar essas coleções e os seus significados obriga a abordagens múltiplas, articuladas e colaborativas, em que a biografia dos objetos e das coleções surge como método fundamental. A análise das proveniências, dos percursos e das relações estabelecidas pelos objetos existentes nas instituições museológicas europeias abre campo para repensar não só práticas museológicas como também a própria construção do conhecimento.

Em Portugal, esse processo está em desenvolvimento em várias instituições museológicas, sendo que neste texto se apresentam alguns resultados em relação às coleções de etnografia do Museu de História Natural e da Ciência da Universidade do Porto (MHNC-UP). Por meio da construção da biografia dos objetos e das coleções pretende-se percecionar os processos de incorporação dessa instituição ao longo do século XX, bem como o papel que essas coleções não europeias tiveram ao longo do tempo.

## “Rose is a rose is a rose”^1^

Um objeto nunca pode ser visto como neutro. Quando um objeto é incorporado num museu, traz consigo uma carga de significados e relações que foram estabelecidas com diferentes atores e contextos ao longo do tempo e, paralelamente, adquire novos significados nessa instituição de conhecimento. Alberti (2005) salienta a importância de referenciar todos esses significados e relações por meio da construção da biografia do objeto, ao mesmo tempo que o coloca num papel fundamental para a compreensão da história das instituições museológicas. As biografias dos objetos são, necessariamente, complexas e dinâmicas, tal como a relação que o visitante estabelece com os objetos de um museu ao longo da permanência destes na instituição, decorrendo esse facto mais das dinâmicas culturais e sociais da comunidade do que do objeto *per si*.

O objeto é visto como um agente ativo, interagindo com o seu entorno. Kopytoff (1986) introduz esse conceito baseado na premissa de que o objeto não é uma entidade isolada e imutável, mas sim uma entidade em constante mutação. Logo, os seus significados – múltiplos, e não único – alteram-se de acordo com os contextos do objeto.

Tendo em conta que a vida de um objeto não se limita a uma simples sucessão de trocas e transações entre proprietários, é fundamental que se estabeleça a sua biografia, que revela também as várias camadas de significados que o objeto acumulou ao longo do tempo, sendo fundamental perceber o seu contexto (Gosden, Marshal, 1999). Os estudos de proveniência potenciam o conhecimento criado a partir de um determinado objeto, uma vez que tentam estabelecer a sua circulação geográfica, a rede de interações entre este objeto e os diferentes contextos históricos que ele atravessou, refletindo as dinâmicas de poder e as estruturas de conhecimento predominantes nos vários momentos da sua vida. É fundamental que se pesquise o contexto de aquisição dos objetos em museus, uma vez que ele condiciona a interpretação que é feita em contexto museológico e o conhecimento produzido sobre eles.

Uma das limitações da biografia dos objetos pode ser a lacuna de informação verificada em algumas instituições museológicas. Por vezes, os dados associados à peça são escassos, o que não permite desenvolver um trabalho de fundo ao nível dos itens ou mesmo das coleções, limitando também a perceção da total diacronia da instituição ao nível da política de incorporações. É ainda fundamental que a documentação utilizada na recuperação da biografia do objeto seja abordada de modo crítico, uma vez que os documentos refletem as ideologias do seu contexto de produção (Duncan, 1995).

Hicks (2020) levanta questões em relação ao foco excessivo unicamente nos objetos, sob o risco de não se conseguir analisar as dinâmicas de poder adjacentes às mudanças de propriedade. O autor propõe, inclusive, que se analise também a constituição das coleções, paralelamente à biografia dos objetos, de modo a percecionar também o impacto das recolhas desses artefactos nas comunidades de origem.

## Em Portugal

Durante 2021, foi direcionado um inquérito do Conselho Internacional de Museus de Portugal (Icom Portugal) a todas as instituições museológicas nacionais, ao qual somente 67 instituições responderam. Para “avaliar a presença de objetos provenientes de contexto extraeuropeu nos museus portugueses”, o resultado deste inquérito (Amaro, Felismino, 2021) surge como um primeiro “retrato” da situação em Portugal. Entre as 67 respostas obtidas, apenas 52 instituições indicam ter coleções não europeias, predominantemente provenientes dos continentes asiático e africano. Interessante é o facto de os museus com números mais elevados de objetos/espécimes provenientes de contextos extraeuropeus serem maioritariamente universitários. Os autores explicam essa situação pelo facto de que os museus universitários “pela sua natureza e história secular de estudo, ensino e prática das ciências, albergam uma grande variedade de coleções de história natural, etnologia e antropologia com números avultados de espécimes” (Amaro, Felismino, 2021, p.129). Sendo esse um bom ponto de partida, é essencial avançar para uma imagem mais nítida da realidade nacional, como os próprios autores indicam. A constituição de coleção nos museus tem, por norma, origens múltiplas e complexas, e o percurso dos objetos e a identificação dos atores envolvidos são fundamentais para a compreensão das dinâmicas das instituições.

O reconhecimento da importância do estudo da proveniência e da biografia dos objetos tem levado a ações de revisão das coleções não europeias. Alguns museus levam já a cabo esse trabalho de compilação de registos e dados associados, com o objetivo de identificar a proveniência dos seus objetos, numa perspetiva de documentação das coleções não europeias e identificação de processos de constituição de coleções. É o caso do Museu Nacional de História Natural e da Ciência da Universidade de Lisboa (Godinho et al., 2021) e do Museu Municipal Santos Rocha – este, por via do projeto Transmat (Ferreira et al., 2021). A reconstituição da biografia e do itinerário geográfico e científico dos objetos surge como fundamental para a compreensão do significado das coleções. As autoras cruzam fontes documentais, históricas, catálogos e registos fotográficos para reconstruir os processos de movimentação de bens culturais que culminam na construção das coleções de comparação.

Uma abordagem idêntica foi realizada por Caldeira (2023) nas coleções etnográficas não europeias do Museu Nacional de Arqueologia, por meio da consulta dos arquivos de inventário com as várias fases de descrição dos objetos, e do Livro de Entradas. A autora salienta, porém, que a pesquisa das proveniências revelou informações incompletas sobre objetos e coleções, ao mesmo tempo que apresenta apenas a perspetiva da potência colonizadora em detrimento do contexto do objeto.

O MHNC-UP também se encontra, neste momento, num processo de revisão das suas coleções arqueológica e etnográfica. Este processo ganha especial significado no âmbito de um potencial levantamento detalhado de coleções não europeias existentes a nível nacional, e a sua disponibilização aos públicos, bem como na identificação de redes de investigação durante o século XIX e XX.

A reestruturação de um museu torna-se um momento único para a transformação de paradigma, em que a valorização da diversidade cultural deve estar na base do processo. Desse modo, a identificação da proveniência e a reconstituição da mobilidade geográfica e cultural do objeto é fundamental.

Nesta pesquisa foram utilizadas as diversas fontes documentais disponíveis, tentando, sempre que possível, cruzar informações, analisando criticamente e contextualizando no tempo os dados disponíveis.

Para tal, foram consultados os catálogos existentes, incluindo as fichas de inventário realizadas a partir do final da década de 1970. Nessas diferentes fases de inventário é possível verificar distintas abordagens ao registo de um objeto, revelando o paradigma cultural e científico a cada época. Foi consultado também o livro “Registo de aquisições”, vigente entre 1926 e 1976 (Registo…, 1926-1976), e listagens elaboradas como o “Cadastro de bens do Instituto de Antropologia”, o processo de transferência dos Museus Estatais de Berlim, os processos de implantação das salas expositivas com as respetivas plantas do edifício e diversos levantamentos realizados ao longo do século XX. A consulta de bibliografia publicada sobre as coleções e pelos investigadores da instituição também foi fundamental, a par de artigos de comunicação social local.

Os objetos foram ainda observados com especial cuidado para a verificação de inscrições e marcações. Em alguns casos, os únicos registos de determinada fase de inventário materializam-se em etiquetas ou numerações aplicadas diretamente nos objetos.

## O Museu de História Natural e da Ciência da Universidade do Porto

O MHNC-UP foi formalmente constituído em 2015. No entanto, a atual instituição constitui-se do culminar de uma sequência de estruturas museológicas preexistentes da Universidade do Porto. Estas incluem os vários museus departamentais criados no seio da Faculdade de Ciências da Universidade do Porto (FCUP) durante a primeira metade do século XX.

Após a criação da Universidade do Porto (instituição de ensino que substituiu a Academia Politécnica), em 1911, são criados laboratórios e museus de apoio às atividades letivas, que estariam à disposição de professores e alunos para a realização de aulas práticas ou aulas de cariz mais expositivo, recorrendo aos vários objetos que constituíam as suas coleções.

Na FCUP são criados, então, o Laboratório e Museu de Antropologia, em 1912, por António Mendes Correia, e os Museus de Zoologia e de Botânica Dr. Gonçalo Sampaio, em 1916. Na década seguinte, em 1923, é criado por decreto-lei (Portugal, 29 dez. 1923) o Instituto de Antropologia, que passa a enquadrar as coleções e atividades de investigação nas áreas de arqueologia, etnografia e antropologia biológica (designada à época como antropologia física). Mais tarde, em 1930, é criado o Museu de Ciências Geológicas.

Apenas no final do século XX, em 1996, os vários museus departamentais da FCUP serão integrados numa única estrutura – o Museu de História Natural da Faculdade de Ciências –, sendo criado no mesmo ano o Museu da Ciência, dedicado sobretudo à instrumentação científica e história da ciência.

O MHNC-UP, atualmente, engloba todas as coleções provenientes dessas estruturas. Integra também as coleções da antiga Academia Politécnica e de outras entidades com atividade no final do século XIX, tais como a Sociedade Carlos Ribeiro.^2^ As incorporações continuam, totalizando o MHNC-UP atualmente mais de oitocentos mil objetos e espécimes das diferentes áreas disciplinares representadas.

Neste artigo trataremos apenas dos objetos e coleções pertencentes ao antigo Museu e Instituto de Antropologia, mais precisamente das coleções de origem não europeia incorporadas durante o século XX. O acervo do antigo Museu de Antropologia está organizado, por tradição, por áreas disciplinares, sendo que dentro dessas podemos encontrar diversas coleções, com uma disposição geográfica prevalecente. Deste modo, as coleções do antigo Museu e Instituto de Antropologia estão catalogadas segundo as áreas disciplinares de arqueologia, etnografia, antropologia biológica^3^ e numismática e medalhística.

Essa classificação por áreas disciplinares ganhará maior expressão no final da década de 1970, coincidindo com uma reorganização das coleções e o início da implementação de um registo ordenado em fichas de papel individualizadas, cumprindo os critérios básicos de inventário propostos pelo International Committee for Documentation (Cidoc-Icom), por meio das 16 categorias gerais de informação para objetos de museu (Chenhall, Homulos, 1978). Desde essa alteração foram feitas algumas atualizações nesse sistema.

Efetivamente, é possível avaliar o modo como os objetos e coleções foram organizados desde a criação do Museu e Laboratório de Antropologia, em 1912. Nos arquivos do MHNC-UP é possível consultar algumas listagens de inventário sumário, bem como o livro de “registo de aquisições”, correspondendo aos registos realizados entre as décadas de 1920 e 1960. Por meio das referidas listagens observa-se uma organização bastante centrada no que se poderia considerar um sistema político-geográfico. Os objetos, todos integrados numa supracategoria – “Antropologia geral e metropolitana” –, estão organizados de acordo com a sua proveniência, ou seja, “Portugal” e “Estrangeiro”. Segue-se um novo nível de organização para os objetos provenientes de Portugal com a associação à sua cronologia, de acordo com as classificações clássicas aplicadas à arqueologia.^4^ O mesmo sistema era aplicado à etnografia, com listagem organizada por “Etnografia geral e metropolitana”, com as mesmas subdivisões “Portugal” e “Estrangeiro”. Num nível seguinte de organização eram descriminados os países estrangeiros dos quais provinham os objetos da coleção.

Após a década de 1970, com a implementação do sistema de inventário seguindo as normativas do Icom, a organização de coleções sofre um pequeno ajuste mais focado na área disciplinar a que pertence o objeto, mantendo, no entanto, esse cariz político-geográfico. Maria José Cunha (2012) menciona a manutenção desse tipo de organização até a primeira década do século XXI. A autora, e anterior curadora das coleções, menciona a utilização de três grandes categorias (dentro de cada área disciplinar) para efeitos de organização e inventário dos objetos. São essas “Portugal”, “Colonial” e “Estrangeira”, sendo que por “Colonial” se entende os objetos com proveniência de Índia, Timor-Leste, Macau, Angola, Guiné, Moçambique e São Tomé e Príncipe (Cunha, 2012). Esse sistema surge ainda como herança do sistema anteriormente existente.

Atualmente, os objetos e coleções estão organizados de acordo com a área disciplinar a que correspondem e, num segundo nível, de acordo com o sítio arqueológico do qual provêm (no caso das coleções arqueológicas) ou a sua proveniência geográfica (no caso das coleções etnográficas), utilizando como referência o tesauro de nomes geográficos do Getty Research Institute (Getty…, 1999).

A implementação de um sistema de gestão de informação multidimensional^5^ permite não só uma organização diferente das coleções, mas também uma incorporação de dados até ao momento lacunares e um cruzamento de informações, potenciando múltiplas abordagens. A indicação dos grupos culturais, bem como das designações originais dos objetos no seu local de origem, tem sido uma aposta do MHNC-UP (ex: Gaspar, Alves, 2022; Santos, 2022). Apesar de se manter uma organização com base na proveniência geográfica dos objetos, atualmente associam-se várias camadas de informação, multiplicando a possibilidade de leituras sobre eles.

## Acervo do Museu de História Natural e da Ciência da Universidade do Porto e coleções não europeias

Como é característico dos museus universitários, as coleções existentes no MHNC-UP apresentam também bastante variabilidade no que se refere a proveniência, tipologia ou mesmo cronologia. Estão representadas culturas dos cinco continentes. O inventário ainda não se encontra finalizado, mas será seguro apontar que o total das quatro áreas disciplinares mencionadas remete para cerca de 125 mil objetos. Mais de 2.500 correspondem a antropologia biológica, cerca de 3.500 a etnografia, mais de 4.300 a numismática e medalhística, e a área mais expressiva é a arqueologia, com mais de 115 mil objetos.

Essas coleções correspondem a duzentos anos de recolha e investigação, permitindo também observar as mudanças de paradigma científico durante esse extenso período. Paradigmas esses também vertidos para a própria organização das coleções nas instituições de memória.

Interessante é também observar como esses objetos e coleções têm sido expostos. Várias fases podem ser documentadas desde o primeiro momento, em 1912, no qual a colocação dos objetos em algumas pequenas salas (Correia, 1941) permitia que os alunos tivessem acesso direto às coleções para estudo. Teríamos, então, uma sala de pré-história, uma sala de etnografia e uma sala de antropologia biológica. Nesse momento, como iremos analisar seguidamente, existe ainda um número reduzido de coleções no museu. Numa fase seguinte, já em meados da década de 1930, uma reorganização do edifício que albergava a FCUP (e onde ainda hoje se encontra o MHNC-UP) permitiu a transferência dos objetos e coleções para duas salas, aumentando grandemente a área expositiva. De salientar que nessa fase os objetos estariam todos expostos, não se tendo documentado a existência de reservas. É também nessa fase que se iniciam as grandes vagas de incorporação de objetos. O Museu de Antropologia passa, então, a ter as suas coleções distribuídas pelas duas salas: a primeira, a Sala Metropolitana, teria, sobretudo, objetos de arqueologia predominantemente provenientes de intervenções no território português, mas também alguns objetos europeus, do Antigo Egipto e da Grécia clássica (Santos Júnior, 1963). Nessa sala estariam ainda itens de etnografia nacional. Na segunda sala, o Museu do Ultramar, estariam peças de etnografia com foco para as coleções provenientes de Guiné, Angola, Moçambique, Timor-Leste, Ásia e Oceânia (Santos Júnior, 1963). A organização dos objetos nas salas seguiria uma lógica cronológica na primeira e uma lógica geográfica na segunda.

Em 1974, inicia-se uma grande alteração. Na sequência do incêndio de 20 de abril desse ano (Fernandes, 2007), o Museu do Ultramar é encerrado, e as coleções aí expostas são colocadas em reserva. Esses objetos não voltariam a ser expostos em permanência, participando apenas em várias exposições temporárias em território nacional e no estrangeiro, nas últimas cinco décadas (cf. Gomes, 2024). Também por essa altura se inicia uma reestruturação da Sala Metropolitana, que abandona essa designação e surge com uma seleção de objetos de arqueologia organizados da seguinte forma: (1) pré-história de Portugal; (2) Paleolítico europeu e africano, arte funerária egípcia e Idade do Ferro da Alemanha; (3) Hominização (Noticiário arqueológico, 1980). São retirados todos os objetos de etnografia, ficando essa disciplina ausente da componente expositiva do Museu de Antropologia. Nessa nova abordagem continua patente o cariz didático e a forte ligação às atividades de ensino desenvolvidas na FCUP.

Após a fusão dos vários museus departamentais no Museu de História Natural da FCUP, em 1996, a sala em questão passa por nova transformação, tornando-se um dos polos do novo museu. A então designada Sala de Arqueologia e Pré-história Mendes Correia abrirá no final de 1998 com uma nova narrativa, apresentando uma seleção de objetos de arqueologia representativos da longa diacronia da ocupação humana do território português. Essa exposição será mantida até 2014.

O MHNC-UP encontra-se atualmente numa fase de reestruturação, tendo sido construídas novas salas de reserva e reestruturadas as existentes (Gaspar, 2020). A implementação no MHNC-UP de um novo sistema de gestão de informação, o In Patrimonium (da empresa portuguesa Sistemas do Futuro), permitiu a associação de múltiplas dimensões de dados ao objeto, potenciando análises transversais não só às coleções do antigo Museu e Instituto de Antropologia, mas também com as restantes coleções de história natural. É de salientar que, nesse contexto universitário, os responsáveis pela incorporação de objetos de arqueologia e etnografia surgem também como coletores para as restantes áreas disciplinares. Nos trabalhos de campo eram muitas vezes recolhidos objetos de arqueologia e/ou etnografia e, paralelamente, espécimes de história natural (botânicos, zoológicos ou mesmo geológicos), que eram então distribuídos pelos diversos departamentos quando os investigadores regressavam à universidade. Esse tipo de gestão da informação em múltiplas dimensões que o MHNC-UP está a implementar permite uma abordagem muito diversificada aos objetos, às coleções e aos atores envolvidos no processo. A possibilidade de vários ângulos de abordagem potencia o incremento do conhecimento sobre as coleções. A construção de biografias dos objetos e a recuperação dos processos e atores envolvidos na constituição das coleções é facilitada. Esse processo tem decorrido cruzando as várias fontes disponíveis, sendo o arquivo do MHNC-UP uma fonte fundamental.

Foi já salientado anteriormente que as coleções de etnografia não europeias deixaram de estar expostas em 1974. Apesar de sempre acessíveis a estudantes e investigadores, é notório que a procura por elas decaiu durante essas cinco décadas. Exceção para a presença em exposições temporárias, cujas solicitações surgem com frequência. Era fundamental reestruturar essa área e potenciar o acesso à informação por parte dos investigadores e especialistas.

O MHNC-UP tem um interessante, ainda que não muito extenso, pelo património de origem não europeia. Em termos gerais, e relativamente a objetos de proveniência não europeia, apenas 14% dos sítios arqueológicos representados no antigo Museu de Antropologia são de origem não europeia. Se olharmos para os objetos etnográficos, a percentagem sobe para cerca de 40%.

Quando analisamos com maior detalhe a proveniência geográfica dos objetos etnográficos não europeus, verificamos que 54% destes têm a sua origem no continente africano, 15% no continente asiático e 12% provêm da Oceânia. Apenas 2% são provenientes do continente americano. Existe ainda um número considerável de objetos (17%) cuja proveniência não foi documentada. A identificação dos países de origem dos objetos pode ser observada na Tabela 1. É de salientar que se utiliza como referência as designações geográficas atuais, pós-coloniais, e não os grupos culturais produtores dos objetos.

**Tabela 1:** : Distribuição geográfica dos objetos arqueológicos e etnográficos de acordo com o país da proveniência

País	Arqueologia	Etnografia
África do Sul	7	
Angola	1	343
Argélia	10	
Benin		6
Camarões		20
Egipto	11	1
Etiópia		1
Guiné-Bissau		103
Marrocos	7	
Moçambique		46
Quénia	5	15
Rep. Democrática do Congo		9
Rep. Popular do Congo		7
Ruanda		2
São Tomé e Príncipe		4
Sudão		2
Tanzânia		13
Zâmbia	2	
Zimbábue	6	2
Brasil	7	2
Colômbia	20	
Costa Rica	1	
México	11	16
Nicarágua	1	
Uruguai		2
China	1	24
Coreia		1
Índia		3
Irão		2
Iraque	4	
Israel	1	
Japão		6
Macau		4
Myanmar		2
Tailândia		3
Timor-Leste		113
Turquia	2	4
Indonésia		14
Papua Nova Guiné		112

Fonte: elaborada pela autora.

No continente africano, predominam claramente os objetos provenientes de Angola, seguindo-se os da Guiné-Bissau e Moçambique. Entre os objetos da Ásia, Timor-Leste domina o conjunto, existindo ainda uma coleção proveniente da Papua Nova Guiné, de dimensão considerável. Se analisarmos a diacronia de incorporação de objetos com proveniência não europeia, as décadas de 1930 e 1940 surgem como as de maior atividade. O museu, criado em 1912, investe no incremento das suas coleções durante as décadas seguintes. Será também interessante perceber que, após esse momento de grande incremento de coleções, as décadas seguintes mostram o movimento inverso. Mendes Correia, o fundador do museu, morre em 1966, e a década de 1970 é marcada por fortes alterações sociopolíticas e pela descolonização. O incêndio de 1974 altera, como já mencionado anteriormente, a dinâmica do museu e relega para segundo plano as coleções de proveniência não europeia. A última incorporação realizada no século XX, em 1997, trata-se de uma transferência de outra instituição da cidade – o Ateneu Comercial do Porto.^6^

Se observarmos a diacronia de incorporação de coleções privadas, vemos que seguem essa tendência geral. No entanto, o contributo dos atores integrados na Universidade do Porto é relativamente constante até a década de 1970. É de salientar que o contributo de outras instituições é pontual, ainda que muito significativo e enriquecedor para essas coleções.

**Tabela 2:** : Diacronia de incorporações no Museu de Antropologia durante o século XX

Institucional										
País	1910’s	1920’s	1930’s	1940’s	1950’s	1960’s	1970’s	1980’s	1990’s	(?)
Angola			1				1		1	
Benim				1						
Camarões				1						
Egito				1						
Rep. Democrática do Congo				1						
Rep. Popular do Congo				1						
Ruanda				1						
Tanzânia				1						
China				1						
Coreia				1						
Índia				1						
Irão				1						
Japão				1						
Myanmar				1						
Tailândia				1						
Turquia				1						
Indonésia				1						
Papua Nova Guiné				1						
**Universidade do Porto**										
**País**	**1910’s**	**1920’s**	**1930’s**	**1940’s**	**1950’s**	**1960’s**	**1970’s**	**1980’s**	**1990’s**	**(?)**
Angola		2		1		3	1			
Moçambique	1			1						
São Tomé e Príncipe					1					
Sudão				1						
Zimbábue			1							
Brasil						2				
Macau						1				
Timor-Leste					1					
**Coleções privadas**										
**País**	**1910’s**	**1920’s**	**1930’s**	**1940’s**	**1950’s**	**1960’s**	**1970’s**	**1980’s**	**1990’s**	**(?)**
Angola	1	1	2							2
Etiópia										1
Guiné-Bissau			3			1				
Moçambique			2							
Quénia			1							
Rep. Popular do Congo										1
Tanzânia			1							
Uruguai			1							
Índia		1								
Japão				1						
Timor-Leste				1		1				
Indonésia			1							

Fonte: elaborada pela autora.

## Identificando e caracterizando os atores no processo

Analisando os dados já tratados relativamente às coleções não europeias de etnografia, podem ser observados três grandes tipos de contributos relativamente às incorporações. Há casos em que, apesar de os objetos terem entrado no museu como coleção privada (ex. de Artur Fonseca Cardoso) ou via transferência de outra instituição (ex. coleção Museus de Berlim, da Faculdade de Letras da Universidade do Porto – FLUP; todo o processo de transferência pode ser consultado em Morais, Gaspar, Reis, 2019), a sua recolha esteve relacionada com programas científicos de trabalho.

Podemos, então, observar que, para além das incorporações realizadas por via de atores integrados na Universidade do Porto, temos a incorporação de coleções por via de transferência de outras instituições e de coleções privadas.

Necessariamente contabilizamos a entrada de objetos por meio de investigadores e colaboradores do Museu e Instituto de Antropologia (ver Quadro 1); no entanto, outras vias de incorporação têm também um enorme peso na constituição dessas coleções. Como referimos anteriormente, outros investigadores e professores da Faculdade de Ciências, de áreas disciplinares distintas, traziam objetos para o museu. Poderemos salientar, a título de exemplo, Américo Pires de Lima (1886-1966), um médico, botânico e professor universitário a quem se deve a incorporação de estatuetas Maconde no primeiro quartel do século XX. Esses atores pertencentes ao universo da Universidade do Porto fizeram importantes contributos para o aumento das coleções de etnografia não europeia.

**Quadro 1:** : Atores envolvidos na incorporação de objetos não europeus no antigo Museu de Antropologia

Contributos para as incorporações no antigo Museu de Antropologia		
Universidade do Porto	Museu/Instituto de Antropologia	António Mendes Correia
		Joaquim Santos Júnior
		António Marques de Almeida Júnior
		João Alves dos Reis Júnior
		António Huet Bacelar Gonçalves
	Faculdade de Ciências	Américo Pires de Lima (Grupos de Zoologia/Botânica)
		Arnaldo Roseira (Grupo de Botânica)
		José Castro Portugal (Mineralogia)
Instituições	Museus de Berlin – FLUP	
	Museu do Instituto Superior do Comércio	
	Instituto de Investigação Científica de Angola	
	Ateneu Comercial do Porto	
Coleções privadas	Carreira administrativa e diplomática	António Pereira Cardoso
		Bernice Bernal
		Humberto Pinto de Lima
		João Graça
	Carreira médica	António Liz Ferreira
		António Nascimento Leitão
	Clero	António Miranda Magalhães
		Claudino Nazareth Brites
	Carreira militar	Afonso Machado de Sousa
		António de Azevedo
		Artur Fonseca Cardoso
		Francisco de Azevedo Gomes
	Outros	–

Fonte: elaborado pela autora.

Outro meio importante de incorporação foram as transferências de coleções de outras instituições, como, por exemplo, do Instituto de Investigação Científica de Angola,^7^ ou mesmo do Ateneu Comercial do Porto. Alguns dos objetos angolanos chegaram via Museu do Instituto Superior do Comércio, criado em 1886 e cujo diretor foi Joaquim de Vasconcelos. Esses objetos corresponderiam às recolhas mais antigas provenientes de Angola existentes no MHNC-UP.

Um interessante exemplo é a coleção Museus de Berlim – FLUP, em que a transferência, em 1927, para a Faculdade de Letras da Universidade do Porto e a posterior incorporação no Museu de Antropologia, em 1940 (Morais, Gaspar, Reis, 2019), incrementam grandemente as coleções etnográficas não europeias. Só essa coleção, que engloba arqueologia e etnografia, aumentou em cerca de setecentos objetos o acervo do antigo Museu de Antropologia. Em termos de etnografia estão representados grupos culturais mexicanos, dos Camarões, do Congo e da Papua Nova Guiné, entre outros. A importância dessa incorporação para o museu é notória nos registos fotográficos na época, nos quais é possível observar o então Museu do Ultramar densamente povoado pelos objetos recebidos da Alemanha.

No entanto, a reconstituição da biografia dos objetos incorporados via outras instituições torna-se mais complexa e dificultada, não havendo garantia da existência de informação associada a eles nas instituições de origem.

As redes de contacto são fundamentais para a incorporação de coleções durante a primeira metade do século XX. Boa parte das coleções identificadas como privadas no Quadro 1 pertencem a coletores cuja ligação ao Museu de Antropologia se faz por meio de colaborações com os investigadores da instituição. Exemplos do reverendo Claudino Nazareth Brites (sócio-fundador da Sociedade Portuguesa de Antropologia e Etnologia, que recolheu um conjunto de objetos em Angola) e do cónego António Miranda Magalhães (que realizava recolhas na Guiné durante o tempo em que foi missionário), cujas coleções foram incorporadas na década de 1930 pelos seus herdeiros. Essa rede de contactos inclui também funcionários administrativos no estrangeiro, diplomatas, médicos e militares. Todos eles recolhem objetos durante os períodos que estiveram fora do país e contribuem para o incremento das coleções não europeias do museu.

Entre as décadas de 1930 e 1960 realizou-se um conjunto de missões antropológicas financiadas pelo Estado português. Foram elas: a Missão Antropológica de Moçambique, entre 1936 e 1956, chefiada por Joaquim Santos Júnior (Santos Júnior, 1938); a Missão Antropológica e Etnológica da Guiné, entre 1946 e 1947, chefiada por Amílcar de Magalhães Mateus; a Missão Antropológica de Timor-Leste, entre 1953 e 1963, chefiada por António de Almeida (Roque, Marques, 2010); e a Missão Antropobiológica a Angola, na década de 1950, chefiada por António de Almeida. Os objetos recolhidos durante as missões a Moçambique e Guiné foram incorporados no Museu de Antropologia aquando das campanhas, tendo sido transferidos para o Instituto de Investigação Científica Tropical em 1988 e 2007, respetivamente. Esse terá sido outro modo de incorporação de coleções não europeias no museu, ainda que esses objetos em particular não pertençam atualmente ao acervo do museu. As coleções foram recolhidas no âmbito de campanhas programadas de investigação coordenadas por investigadores do Instituto de Antropologia. São casos únicos no que refere a etnografia não europeia, uma vez que a investigação programada no instituto se centrou, sobretudo, em temáticas e contextos nacionais.

## Considerações finais

O trabalho desenvolvido até ao momento com as coleções não europeias do antigo Museu de Antropologia permite percecionar as tendências gerais de incorporação, bem como identificar os principais atores envolvidos. A identificação da tendência crescente de integração de coleções durante as décadas de 1930 e 1940 e do sucessivo decréscimo na segunda metade do século XX parece alinhar-se com o contexto sociopolítico de Portugal e com o investimento realizado a nível científico nessas áreas específicas. Salienta-se que, no caso do antigo Museu de Antropologia, a incorporação de coleções particulares é bastante considerável. Os vários atores identificados no processo de incorporação de objetos não europeus acabam por aumentar o espólio do antigo Museu de Antropologia por via de recolhas realizadas durante as suas atividades profissionais no estrangeiro.

No processo de levantamento biográfico dos objetos, a sua musealização surge como um dos momentos importantes, fundamental também na compreensão das dinâmicas das instituições de memória. O levantamento das distintas fases existentes ao nível do registo e gestão de coleções no MHNC-UP permite também identificar alterações ao nível da abordagem a esses objetos. A análise das distintas configurações e narrativas nas salas de exposição ao longo do século XX, até a retirada dos objetos de exposição, é também fundamental e elucidativa do seu papel no tecido social. Neste momento, o MHNC-UP encontra-se em reestruturação e em redefinição das suas salas expositivas, sendo que a introdução de novas narrativas é fundamental, de modo a permitir ao visitante múltiplas e descentradas leituras.

## NOTAS

1 Do poema “Sacred Emily”, de Gertrude Stein (1922).

2 A Sociedade Carlos Ribeiro foi fundada em 1887, na cidade do Porto, por cinco estudantes da Academia Politécnica: António Augusto da Rocha Peixoto, Ricardo Severo da Fonseca Costa, Artur da Fonseca Cardoso, Alfredo Xavier Pinheiro e João Barreira. A designação pretendia prestar homenagem ao geólogo e arqueólogo Carlos Ribeiro, pelo seu importante trabalho em Muge sete anos antes. A Sociedade publicou, entre 1889 e 1898, a *Revista de Sciencias Naturaes e Sociaes*, meio de divulgação dos trabalhos realizados em arqueologia, etnografia e antropologia.

3 Esta área disciplinar foi criada em 2016, aquando do início do processo de revisão de coleções no MHNC-UP, para integrar todos os elementos ósseos humanos do século XIX e XX utilizados como coleção de referência e em contexto de ensino da medicina e antropologia, e que estavam anteriormente junto com as coleções de arqueologia.

4 O sistema das três idades, desenvolvido por Christian Jürgensen Thomsen no século XIX e aplicado à arqueologia pré-histórica europeia, está na base da classificação por complexos cronotecnológicos das realidades arqueológicas.

5 Este sistema de gestão de informação está a ser implementado desde 2020 nas coleções de arqueologia, etnografia e antropologia biológica.

6 O Ateneu Comercial do Porto foi fundado em 1869, por um grupo de cidadãos portuenses, com o objetivo de promover o desenvolvimento cultural, científico e social. Esse importante ponto de encontro na cidade desenvolveu inúmeros encontros literários, palestras e debates. É detentor de um importante acervo artístico e documental.

7 O Instituto de Investigação Científica de Angola (atual Centro Nacional de Investigação Científica) foi criado pelo decreto 40.078, de 7 de março de 1955, a par com o Instituto de Investigação Científica de Moçambique, com o objetivo de fomentar a cultura e promover a investigação científica no território ultramarino nas áreas de geografia, história natural e ciências humanas.
